# Screening of lncRNA-miRNA-mRNA Coexpression Regulatory Networks Involved in Acute Traumatic Coagulation Dysfunction Based on CTD, GeneCards, and PharmGKB Databases

**DOI:** 10.1155/2022/7280312

**Published:** 2022-04-19

**Authors:** Yong Luo, Yong Fu, Taifa Tan, Jun Hu, Fang Li, Zhanchen Liao, Jian Peng

**Affiliations:** ^1^Hengyang Medical School, University of South China, Hengyang 421001, China; ^2^Critical Care Medicine & Trauma Centre, The Second Affiliated Hospital of the University of South China, Hengyang 421000, China; ^3^Trauma Orthopedic Department & Trauma Centre, The Second Affiliated Hospital of the University of South China, Hengyang 421000, China; ^4^Radiology Department & Trauma Centre, The Second Affiliated Hospital of the University of South China, Hengyang 421000, China; ^5^Cardiothoracic Surgery & Trauma Centre, The Second Affiliated Hospital of the University of South China, Hengyang 421000, China; ^6^Urology Surgery & Trauma Centre, The Second Affiliated Hospital of the University of South China, Hengyang 421000, China; ^7^Department of General Surgery, Xiangya Hospital, Central South University, Changsha 410008 P. R., China

## Abstract

Competitive endogenous RNA (ceRNA) networks play crucial roles in multiple biological processes and development of diseases. They might serve as diagnostic and prognosis markers as well as therapeutic targets. The purpose of this study was to identify a novel ceRNA network involving KCNQ1OT1, hsa-miR-24-3p, and VWF in acute traumatic coagulopathy (ATC) based on databases search. We searched the CTD, GeneCards, and PharmGKB databases for ATC-related target genes using Coagulopathy as a keyword. Upstream miRNAs and lncRNAs of the candidate target VWF were then explored using the miRWalk, microT, TargetScan, RNA22 and Tarbase, and DIANA-LncBase and Starbase databases, respectively. A KCNQ1OT1-hsa-miR-24-3p-VWF ceRNA network was constructed by *R* “ggalluvial” package. Interaction between KCNQ1OT1, hsa-miR-24-3p, and VWF was examined, and their expression was quantified in the peripheral blood samples from 30 ATC patients and liver tissues of ATC rat models. Forty-one ATC-related target genes were identified following data retrieval from publicly available databases, of which VWF was selected as the target and used for the subsequent analysis. KCNQ1OT1 and hsa-miR-24-3p were confirmed to be the key upstream regulatory factors of VWF. KCNQ1OT1-hsa-miR-24-3p-VWF coexpression regulatory network was constructed where KCNQ1OT1 competitively bound to hsa-miR-24-3p and attenuated its binding to VWF. Both the liver tissues of ATC rats and peripheral blood samples from ATC patients showed increased hsa-miR-24-3p expression and decreased VWF and KCNQ1OT1 expression. Collectively, we described the KCNQ1OT1-hsa-miR-24-3p-VWF ceRNA network in the development of ATC. We propose a new ceRNA that could help in the diagnosis and treatment of ATC.

## 1. Introduction

Despite improvements in the care of trauma patients, the onset of coagulopathy exacerbates hemorrhaging and dramatically increases mortality [1]. Acute traumatic coagulopathy (ATC) represents a failure of coagulation homeostasis that can rapidly occur following traumatic injury, hemorrhage, and shock [2]. Early identification of patients with ATC would be valuable to facilitate appropriate management [3]. The underlying mechanisms of ATC remain under active investigation due to the large number of symptoms, phenotypes. and altered states in the ATC patients [4].

The long noncoding RNA-microRNA-messenger RNA (lncRNA-miRNA-mRNA) competing endogenous RNA (ceRNA) network provides a better understanding of disease pathogenesis and facilitates disease diagnosis and treatment strategies [5, 6]. Von Willebrand Factor (VWF) is a large glycoprotein which mediates platelet adhesion to the subendothelium during vascular injury and plays an important role in hemostasis and thrombosis [7]. Deficiency of VWF can cause enhanced vascularization, both constitutively and following ischemia and meanwhile, this deficiency leads to impaired arteriogenesis and angiogenesis following ligation of the femoral artery [8]. A previous study reported that miR-24 could negatively regulate the expression of VWF by directly binding to the 3′untranslated region (3′UTR) of VWF [9]. miR-24-3p can serve as a promising biomarker to predict the worse prognosis of patients owing to its significant correlation to the recurrence-free survival and disease-free survival [10]. miR-24-3p is often regulated the upstream lncRNAs [11, 12].

Such a lncRNA, lncRNA KCNQ1OT1 has been implicated in many types of human diseases; specifically, KCNQ1OT1 was significantly upregulated in ischemic stroke, and its knockdown reduces infarct volume and neurological impairments in transient middle cerebral artery occlusion (tMCAO) mice [13]. KCNQ1OT1 facilitates the progression of acute myeloid leukemia, as shown by increased proliferation, migration, and invasion of acute myeloid leukemia cells [14]. In the current study, we combined data retrieval from publicly available databases and functional assays to identify the candidate target genes, miRNAs and lncRNAs related to ATC, and in addition to their expression, their associated regulatory network was investigated. After important ATC-related candidates were identified, we established a KCNQ1OT1-hsa-miR-24-3p-VWF ceRNA regulatory network that can elucidate the underlying etiology of ATC and further provide molecular targets for treatment.

## 2. Materials and methods

### 2.1. Ethics Statement

The current study was approved by the Ethics Committee of the Second Affiliated Hospital of the University of South China and performed in strict accordance with the *Declaration of Helsinki*. All participants and/or their caregivers signed informed consent documentation. Animal experiments were performed with the approval of the Ethics Committee of the Second Affiliated Hospital of the University of South China and performed in strict accordance with the Guide for the Care and Use of Laboratory animals published by the US National Institutes of Health.

### 2.2. Databases' Search

ATC-related target genes were retrieved in CTD (Inference score ≥30; http://ctdbase.org/) and GeneCards (Relevance score ≥0.21; https://www.genecards.org/) databases using “Coagulopathy” as a keyword. Draw Venn Diagram tool (http://bioinformatics.psb.ugent.be/webtools/Venn/) was applied for intersection analysis of the results from the two databases. Disease targets were identified using the PharmGKB database (https://www.pharmgkb.org/). Next, the interaction network of target genes was obtained using the STRING database (https://string-db.org), with the species set as “Homo sapiens,” which was further introduced into the Cytoscape software to construct the regulatory network. Meanwhile, the interaction network was analyzed by Cytoscape, and the results were screened. The degree value and combine score value were expressed by color scale. Thereafter, the candidate target genes related to ATC were subjected to Gene Ontology (GO) and Kyoto Encyclopedia of Genes and Genomes (KEGG) enrichment analyses using the *R* “ClusterProfiler” package (http://www.bioconductor.org/packages/release/bioc/html/clusterProfiler.html).

Upstream miRNAs of the candidate target gene VWF were predicted using the miRWalk (score>0.8; http://mirwalk.umm.uni-heidelberg.de/), microT (miTG score>0.998; http://diana.imis.athena-innovation.gr/DianaTools/index.php?r=microtv4/index), TargetScan (species = human, conserved microRNA family = all; http://www.targetscan.org/mamm_31/), RNA22 (sensitivity of 63%, specificity of 61%; http://cbcsrv.watson.ibm.com/rna22.html), and Tarbase databases (methods = IP; http://microrna.gr/tarbase/), followed by intersection analysis. The upstream lncRNAs of the predicted miRNA were predicted by employing the DIANA-LncBase (score = 1.0, threshold > 0.9; http://carolina.imis.athena-innovation.gr/diana_tools/web/index.php?r=lncbasev2/index-predicted) and Starbase (genome = human, number of supporting experiments > 1, target sites > 1; http://starbase.sysu.edu.cn/starbase2/). Finally, the lncRNA-miRNA-VWF coexpression regulatory network was constructed, and Sankey diagram analysis was conducted using the *R* “ggalluvia” package.

### 2.3. Sample Collection

Thirty patients with ATC referred to the Department of Emergency of The Second Affiliated Hospital of the University of South China were recruited in the current study. Meanwhile, thirty healthy individuals receiving physical examination at The Second Affiliated Hospital of the University of South China during the same period were selected as the control. Next, 5 mL of peripheral blood samples was collected from the included subjects and stored in a -80°C freezer, followed by examination of blood routine (PLT and PT), coagulation index (APTT and INR), and six new thrombus items (TAT, PIC, TM, t-PAIC, FDP, and D-dimer).

### 2.4. Establishment of ATC Rat Models

Thirty rats were randomly divided into an ATC group and a control group, with 15 rats in each group. The rats were injected intraperitoneally with 1% pentobarbital sodium at a dose of 35 mg/kg for general anesthesia, and the body temperature was maintained at (37 ± 1)°C. Tracheotomy and intubation were performed to assist breathing. A polyethylene latex tube was inserted into the left carotid artery and connected to a pressure sensor and syringe pump. The catheter was flushed to keep the ductus arteriosus unobstructed. The control rats were only anesthetized, intubated, and monitored for blood pressure. The bilateral tibia and fibula of ATC rats were clipped with surgical forceps. The trauma was completed immediately after carotid catheterization. Blood-letting was then conducted to enable the mean arterial pressure reduce to 40-50 mmHg (1 mmHg = 0.133 kPa) within 10 min and maintained. The blood pressure of rats was monitored. The arterial blood sample was extracted after arterial catheterization (T1), at shock (*T* = 0 min; T2) and 60 min (T3), 180 min (T4), and 240 min (T5) after shock. The prothrombin time (PT) and activated partial thromboplastin time (APTT) in the serum were measured. After 240 min, 2 mL of blood sample was drawn from the artery catheter to prepare plasma. The remaining was centrifuged at 1000 g and 4°C for 15 min, and the supernatant was harvested and stored at -80°C. The rats were euthanized, and the pathological tissues were analyzed using Hematoxylin-eosin (HE) staining. The tissue homogenate was prepared and centrifuged at 5000 g and 4°C for 10 min, with the supernatant obtained and stored at -80°C.

### 2.5. PT and APTT Measurement

For PT measurement, the fasting venous blood was drawn with a silicified or plastic syringe, added to silicified or plastic tubes containing 0.109 mol/L sodium citrate anticoagulant at a ratio of 9 : 1 and mixed gently. The mixture was then centrifuged at 2000 - 2500 g for 15 with, followed by isolation of platelet-rich plasma. The measurement temperature was 36.5-38.5°C, and the above reagents and the plasma tested should be preheated to this temperature. However, the pretemperature of thromboplastin reagent should not exceed 30 min, and that of plasma should not exceed 10 min. All the instruments involving plasma, such as test tubes and samplers, were plastic or silicified glass tubes. Next, 0.1 mL of citrate anticoagulant plasma sample was drawn and added to a small tube, which was mixed with 0.1 mL of thromboplastin reagent and incubated in a 37°C water bath. Afterwards, 0.1 mL CaCl_2_ (25 mmol/L) was added to the sample, or thromboplastin reagent was mixed with equal amount of CaCl_2_ and subsequently mixed. The stopwatch was turned on. The tube was still immersed in the water bath, and until about 10 s, it was taken out from the water bath. The water drop outside the tube was wiped quickly using gauze and at the bright place outside, the tube was tilted continuously, whereupon fibrin formation was observed in the flowing state. Once fibrin was visible (liquid flow slowed down at the same time), the watch was stopped, and the time was recorded. Two tubes were measured at a time and reported on average. The pH value of the final mixture of this test should be 7.2-7.3. Most commercial thromboplastin reagents were prepared with buffer solution. Negative and positive controls were set for each batch simultaneously, and the method should be identical with the test specimen.

For APTT measurement, the sample collection, storage and transportation, and the preparation of samples (platelet-free plasma) were the same as those for PT measurement. Clean plastic or silicified glass blood sampler shall be used for blood collection and blood storage. One copy of APTT reagent preheated not more than 30 min was mixed with one copy of plasma to be tested preheated not more than 10 min, and the stopwatch was turned on. At the end of the specified contact activation time, a portion of CaCl_2_ solution preheated to 37°C was added and mixed, with the stopwatch opened at the same time. Upon plasma coagulation, the watch was stopped, and the plasma coagulation time was recorded in seconds. In manual measurement, two tubes should be measured at the same time and reported according to the average. Some automatic or semiautomatic coagulometer with greatly improved precision should be determined only once if there was an appropriate quality control standard. Negative and positive control plasma should be determined concomitantly.

### 2.6. HE Staining

The rat tissues of small intestine, liver, and skeletal muscle were fixed with 10% neutral formalin, paraffin-embedded, and cut into sections. The sections were then dewaxed, hydrated, and cleared with in xylene and rehydrated in descending series of alcohol. Following washing using distilled water, the sections were stained with Harris hematoxylin for 3-8 min, washed with tap water, differentiated with 1% hydrochloric acid-ethanol for several seconds, and blued in 0.6% ammonia water. Following tap water washing, the sections were stained with eosin for 1-3 min, dehydrated, and cleared in xylene. Thereafter, the sections were dried slightly, mounted with neutral gum, and observed under a microscope, with images captured for analysis.

### 2.7. RNA Isolation and Quantitation

Total RNA was extracted from the peripheral blood of rats with TRIzol Reagents (Invitrogen Inc., Carlsbad, CA, USA) and then reverse transcribed into complementary DNA (cDNA) as per the instructions of the TaqMan microRNA Assays Reverse Transcription Primer (4427975, Applied Biosystems, Carlsbad, CA, USA). Reverse transcription quantitative polymerase chain reaction (RT-qPCR) was conducted to quantify the expression of target genes. The primers were designed using the primer design function in the National Center for Biotechnology Information (NCBI) database and are shown in Supplementary Table 1. *β*-Actin served as a loading control for mRNA, and the fold changes were calculated using relative quantification (the 2^-*ΔΔ*Ct^ method).

### 2.8. Western Blot Analysis

Total protein was extracted from the peripheral blood cells of rats with kits (BB-3121, Shanghai Best Biotechnology Co., Ltd., Shanghai, China), with the concentration determined by a bicinchoninic acid (BCA) kit (20201ES76, YEASEN Biotechnology Co., Ltd., Shanghai, China). The protein (20 *μ*g) was then separated using 8% sodium dodecyl sulfate-polyacrylamide gel electrophoresis and transferred onto polyvinylidene fluoride (PVDF) membranes. The membranes were blocked using 5% skimmed milk powder and underwent overnight incubation at 4°C with primary antibodies against *β*-actin (ab8227, 1 : 1000, Abcam Inc., Cambridge, UK) (ab174294, 1 : 10000, Abcam) and VWF (ab28482, 1 : 500, Abcam). The next day, the membranes were incubated with horseradish peroxidase-labeled secondary antibody goat anti-mouse (ab6789, 1 : 1000, Abcam) at room temperature for 1 h. The immunocomplexes on the membrane were visualized using enhanced chemiluminescence (ECL) reagent (ECL808-25, Biomiga Inc., USA), and band intensities were quantified using ImageJ software. The ratio of the gray value of the target band to that of *β*-actin was representative of the relative protein expression.

### 2.9. Dual-Luciferase Reporter Assay

The 3′UTR gene fragment of VWF was cloned and amplified. The PCR product was cloned into the polyclonal site downstream of luciferase gene pmirGLO (E1330, Promega Corporation, Madison, WI), named pVWF-wild type (WT). Site-directed mutagenesis was performed in the predicted hsa-miR-24-3p binding site in the 3′UTR of target gene by TargetScan database analysis to construct pVWF-mutant type (MUT) vector. The pRL-TK vector (E2241, Promega) expressing renilla luciferase was regarded as the loading control. hsa-miR-24-3p mimic and NC mimic were cotransfected into HEK293 cells with luciferase reporter vectors. The luciferase activity was measured by the method provided by Promega. The targeting between KCNQ1OT1 and hsa-miR-24-3p was identified using the same method.

### 2.10. RNA Binding Protein Immunoprecipitation (RIP) Assay

RIP assay was conducted using magna RIP TM RIP Kit (Millipore, Billerica, MA, USA). Peripheral blood cells of ATC rats were lysed in complete RNA lysis buffer, after which the cell lysate was supplemented with RIP immunoprecipitation buffer containing negative control IgG or AGO2 antibody (mouse, Millipore) coupled magnetic beads and incubated overnight. The next day, the sample was incubated with proteinase K for 30 min, and the immunoprecipitated RNA was extracted. Finally, the expression of KCNQ1OT1 and hsa-miR-24-3p was detected by RT-qPCR and agarose gel electrophoresis.

### 2.11. RNA Pull-Down Assay

The ceRNA network between KCNQ1OT1 and miR-24-3p was analyzed by the RNA pull-down assay. The peripheral blood cells of ATC rats were lysed and incubated with biotin-coupled hsa-miR-24-3p probe prebound to the magnetic beads. The target RNA was extracted using the RNeasy Mini Kit (Qiagen company, Hilden, Germany) for 2 h. The pulled down product was extracted, and the expression of KCNQ1OT1 was detected by RT-qPCR.

### 2.12. Statistical Analysis

All data were analyzed using SPSS 21.0 statistical software (IBM Corp. Armonk, NY, USA). The measurement data were described as mean ± standard deviation. Data between two groups were compared by unpaired *t*-test. Differences among multiple groups were statistically analyzed employing one-way analysis of variance (ANOVA), followed by Tukey's post hoc tests with corrections for multiple comparisons. Bonferroni-corrected repeated measures ANOVA was used to compare data at different time points. A value of *p* < 0.05 was statistically significant.

## 3. Results

### 3.1. VWF Is a Potential Key Gene Involved in the Development of ATC

**Figure 1:**
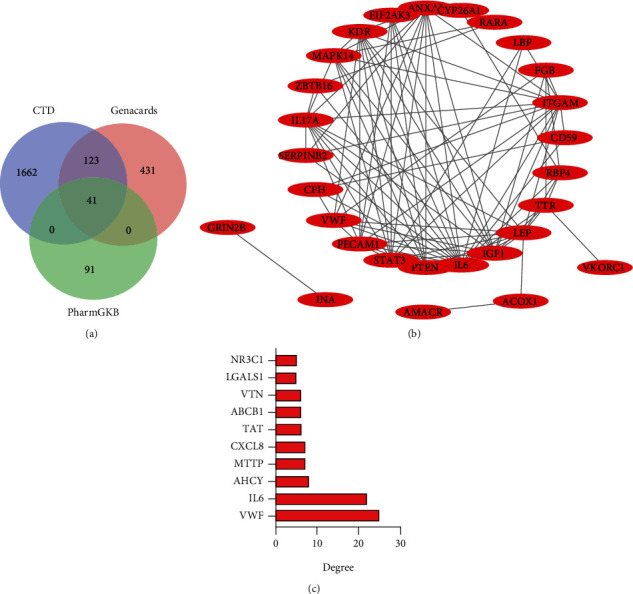
Screening of ATC-related target genes. (a) Venn diagram analysis of genes related to ATC predicted by the CTD and GeneCards databases. (b) A protein-protein interaction network of 41 candidate disease targets. Node represents protein, and edge represents the correlation between proteins. (c) A diagram of 41 candidate disease targets sorted by degree.

The CTD database retrieved 1826 target genes related to ATC, and the GeneCards database retrieved 595 target genes. Following Venn diagram analysis, a total of 164 genes were found at the intersection. The 164 genes and 132 ATC-related target genes obtained from the PharmGKB database were subjected to Venn diagram analysis, which revealed 41 candidates, including IL17A, ITGAM, CYP26A1, SLC4A1, IGF1, SERPINB2, MAPK14, and KDR (Figure 1(a)). A protein-protein interaction network associated with the 41 candidates was then constructed (Figure 1(b)).

After trauma, coagulation components such as fibrinogen and other coagulation factors in the blood will decrease with the blood loss and consumption caused by coagulation activation, resulting in ATC [15], with platelet aggregation in situ as an activated coagulation cascade, a stable blood clot formations [16]. VWF can affect coagulation function by influencing the activity of coagulation factor VIII. If there is not enough functional VWF, platelet adhesion and aggregation will be damaged, the level of VIII decreases, and the time for both blood clot formation and bleeding is prolonged [17]. In this study, the screening results of disease databases also showed that VWF was closely related to ATC (candidate target gene degree ranked the first; Figure 1(c)). Therefore, we chose VWF as the target gene for the follow-up study.

### 3.2. Forty-One ATC-Related Target Gene Function Mainly in Platelet Formation and Fibrinogen Consumption

**Figure 2:**
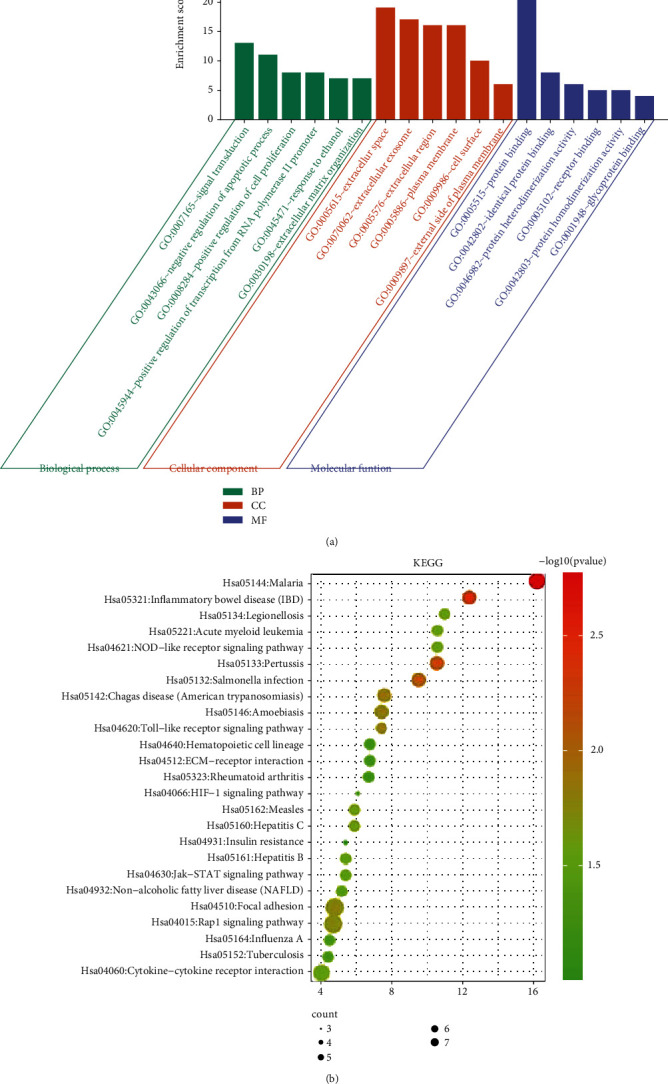
Enrichment analysis of 41 candidate target genes. (a) GO enrichment analysis of 41 candidate target genes. (b) KEGG enrichment analysis of 41 candidate target genes. The dot size indicates the number of selected genes, and the color represents the *p* value of enrichment analysis.

Forty-one candidate target genes related to ATC were subjected to GO and KEGG enrichment analyses. The results of biological process (BP) analysis showed that these target genes were mainly enriched in bone mineralization involved in bone maturation (GO:0035630), T-helper 17 cell lineage commitment (GO:0072540), and negative regulation of myelination (GO:0031642). Cellular component (CC) analysis indicated that they were predominantly enriched in platelet alpha granule (GO:0031091), platelet alpha granule lumen (GO:0031093), and external side of plasma membrane (GO:0009897). In addition, molecular function (MF) analysis suggested that they were chiefly enriched in retinoic acid binding (GO:0042974), complement binding (GO:0001848), retinoid binding (GO:0005501), and Hsp90 protein binding (GO:0051879) (Figure 2(a)). These results demonstrated that 41 candidate target genes play major roles in myelination and platelet formation and were enriched in cell signal receptor and cell membrane structure. Their molecular functions were mainly involved in cell associated protein binding and receptor activity regulation.

KEGG enrichment analysis revealed these target genes were principally distributed in ubiquinone and other terpenoid-quinone biosynthesis (map00130), complement and coagulation cascades (map04610), malaria (map05144), EGFR tyrosine kinase inhibitor resistance (map01521), acute myeloid leukemia (map05221), and inflammatory bowel disease (map05321) (Figure 2(b)). Overall, these findings support that the 41 candidate target genes mainly function in the platelet formation and fibrinogen consumption.

### 3.3. KCNQ1OT1 and hsa-miR-24-3p Are the Key Upstream Regulatory Factors of VWF, Involved in the Development of ATC

**Figure 3:**
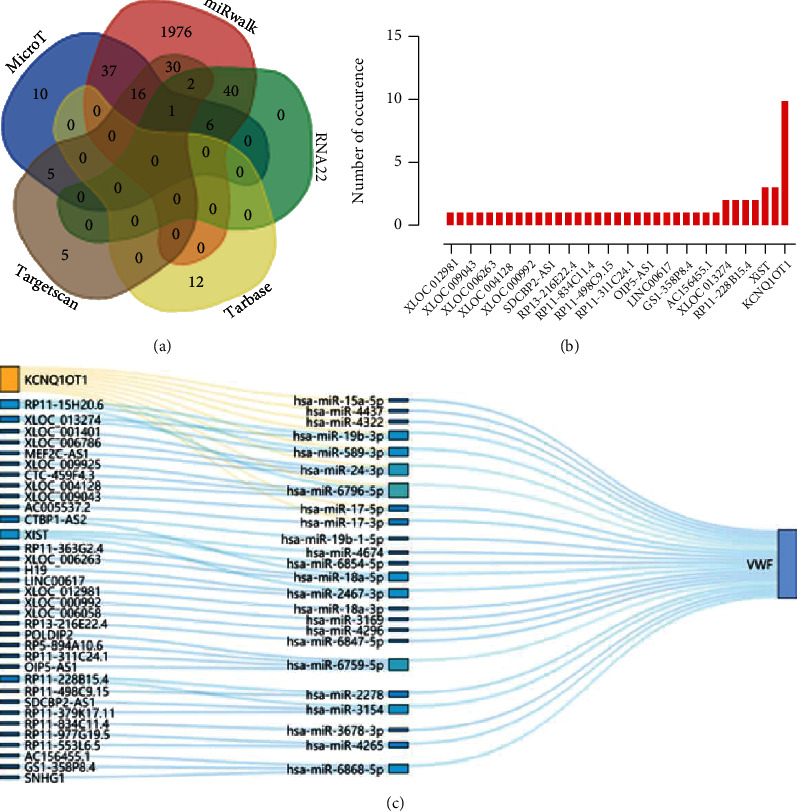
ceRNA network involved in the development of ATC. (a) Upstream miRNAs of VWF predicted by the TargetScan, MicroT, miRWalk, RNA22, and Tarbase databases. (b) Ranking of the predicted lncRNAs. The ordinate represents the predicted number of occurrence (higher frequency of occurrence for a gene reflects closer relationship with the disease). (c) Sankey diagram of the lncRNA-miRNA-VWF network plotted by *R* “ggalluvial” package (higher rectangle reflects higher frequency of its occurrence as a target).

We then attempted to analyze the upstream miRNAs of VWF. The TargetScan, MicroT, miRWalk, RNA22, and Tarbase databases predicted 61, 82, 4104, 103, and 21 miRNAs, respectively. These miRNAs were subjected to intersection analysis (Figure 3(a)), the results of which showed 25 miRNAs at the intersection of three or more databases and hsa-miR-24-3p existed in four databases (Supplementary Table 2). hsa-miR-24-3p was thus used for the subsequent analysis.

DIANA-LncBase and StarBase databases predicted the upstream lncRNAs of 25 miRNAs, and 35 candidate lncRNAs were found at the intersection (Supplementary Table 3). Sankey diagram of the lncRNA-miRNA-VWF network plotted by *R* “ggalluvial” package displayed that KCNQ1OT1 had targeting relationship with multiple miRNAs (Figures 3(b) and 3(c)). Thus, KCNQ1OT1 was used as the target lncRNA for subsequent research. These results suggested that KCNQ1OT1 and hsa-miR-24-3p were the key upstream regulatory factors of VWF, and the coexpression regulatory network of KCNQ1OT1-hsa-miR-24-3p-VWF may be essential for the development of ATC.

### 3.4. KCNQ1OT1 Upregulates VWF Expression by Competitively Binding to Hsa-miR-24-3p

**Figure 4:**
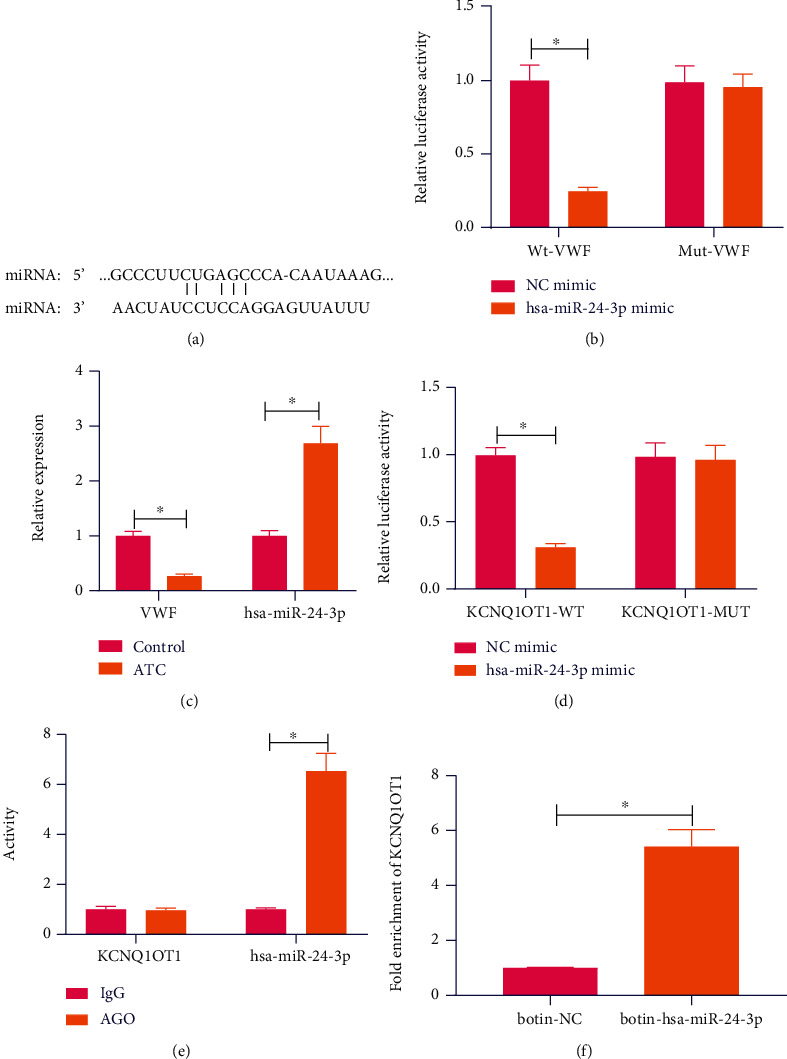
KCNQ1OT1 increases the VWF expression by competitively binding to hsa-miR-24-3p. (a) Binding sites of hsa-miR-24-3p in the 3′UTR of VWF predicted by the TargetScan database. (b) Binding of hsa-miR-24-3p to VWF confirmed by dual-luciferase reporter assay. (c) hsa-miR-24-3p and VWF expression in tissues of control and ATC rats determined by RT-qPCR. (d) Binding between hsa-miR-24-3p and KCNQ1OT1 analyzed by dual-luciferase reporter assay. (e) Binding between hsa-miR-24-3p and KCNQ1OT1 analyzed by RIP assay. (f) Binding between hsa-miR-24-3p and KCNQ1OT1 analyzed by RNA pull-down assay.

We compared the clinical characteristics of the included ATC patients and healthy individuals and found no significant difference in gender and age between the two groups of subjects while blood routine, coagulation indicators, and six new thrombus items were statistically significant (Supplementary Table 4). The TargetScan database predicted the binding sites of hsa-miR-24-3p in the 3′UTR of VWF (Figure 4(a)). Meanwhile, dual-luciferase reporter assay demonstrated that transfection with hsa-miR-24-3p mimic in 293T cells decreased the luciferase activity of VWF-WT without altering the luciferase activity of VWF-MUT (Figure 4(b)). RT-qPCR results showed higher hsa-miR-24-3p expression and lower VWF expression in tissues of ATC rats than control rats (Figure 4(c)). These results suggested that hsa-miR-24-3p could target VWF and inhibit its expression.

Additionally, the luciferase activity of KCNQ1OT1-WT was found to be inhibited in 293T cells transfected with hsa-miR-24-3p mimic while that of KCNQ1OT1-MUT showed no changes (Figure 4(d)), indicating the interaction between hsa-miR-24-3p and KCNQ1OT1. Furthermore, RIP data presented that both KCNQ1OT1 and hsa-miR-24-3p could bind to AGO2 (Figure 4(e)) and meanwhile, RNA pull-down assay results showed that hsa-miR-24-3p could pull down KCNQ1OT1 (Figure 4(f)). This suggested that KCNQ1OT1 targeted hsa-miR-24-3p and suppressed its expression. Taken together, these lines of evidence indicated that KCNQ1OT1 might competitively bind to hsa-miR-24-3p and cause upregulation of the hsa-miR-24-3p target VWF.

### 3.5. KCNQ1OT1-hsa-miR-24-3p-VWF ceRNA Network Closely Relates to the Development of ATC

**Figure 5:**
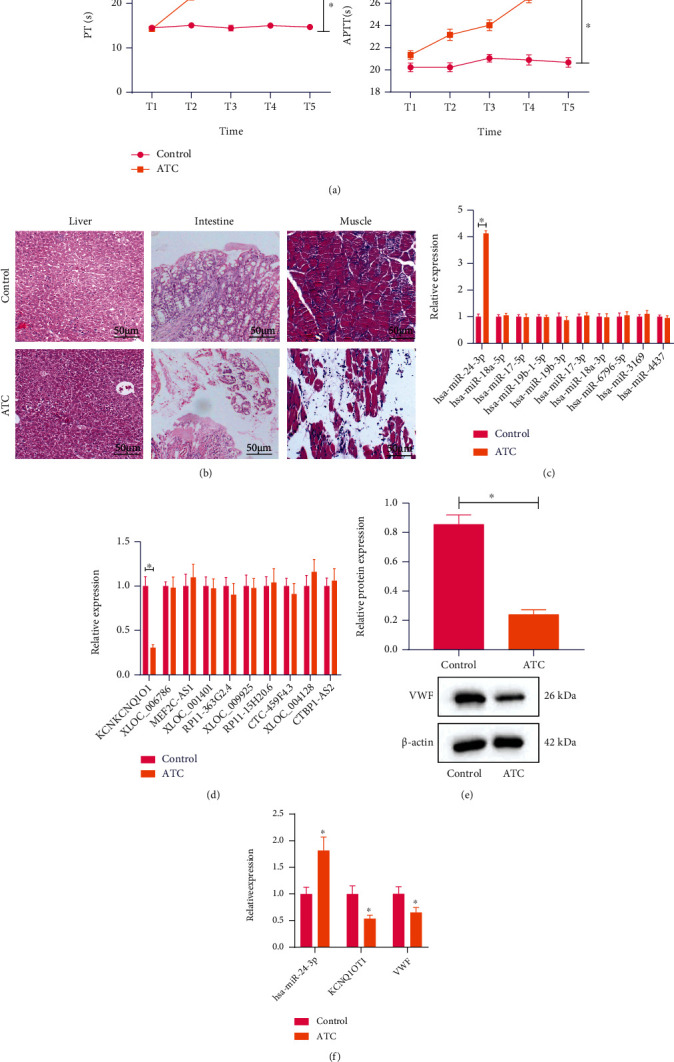
KCNQ1OT1-hsa-miR-24-3p-VWF ceRNA network shows a strong correlation with the development of ATC. (a) PT and APTT of ATC and control rats at different time points. (b) HE staining analysis of small intestinal tissues, liver tissues, and skeletal muscle tissues of ATC and control rats. (a) indicates the liver tissues of control rats, (b) indicates the liver tissues of ATC rats, (c) indicates the mall intestinal tissues of control rats, (d) indicates the mall intestinal tissues of ATC rats, (e) indicates the skeletal muscle tissues of control rats, and (f) indicates the skeletal muscle tissues of ATC rats. (c) Expression of the top 10 candidate miRNAs in the liver tissues of ATC rats examined by RT-qPCR. The abscissa represents the name of miRNAs, and the ordinate represents the expression. (d) Expression of the top 10 candidate lncRNAs in the liver tissues of ATC and control rats examined by RT-qPCR. The abscissa represents the name of lncRNAs, and the ordinate represents the expression. (e) Western blot analysis of VWF protein in the liver tissues of ATC and control rats, normalized to *β*-actin. (f) Expression of KCNQ1OT1, hsa-miR-24-3p, and VWF in the peripheral blood of ATC patients and healthy individuals examined by RT-qPCR. The experiment was conducted three times independently.

An ATC rat model was constructed after which the PT and APTT of ATC and control rats at different time points were measured. The results showed that PT and APTT of ATC rats were higher than control rats (Figure 5(a)). HE staining of liver tissues suggested clear structure of lobules, hepatic cords, sinuses and portal areas, the cord-like hepatocytes with order arrangement, and slit-shaped hepatic sinusoid in the control rats. In contrast, the ATC rats exhibited mild swelling and loosening of some hepatocytes, formation of a few vacuoles, mild dilation of some blood vessels and sinusoid, and formation of hemorrhagic foci.

Moreover, analysis on the small intestinal tissue by HE staining showed that the structure of small intestinal tissues was contact, the mucosal glands were arranged tightly and continuously, and the morphology was normal without bleeding, erosion, or inflammatory cell infiltration in the control rats. According to Chiu's small intestinal injury degree score, the histopathological score of control rats was 0. In ATC rats, the structure of villi in many places of small intestinal mucosa was broken and disordered, and a large amount of red blood cell bleeding was visible in submucosa, with the histopathological score of 5. In addition, HE staining results of skeletal muscle tissues showed that the structure of skeletal muscle cells was contact and clear, and the muscle cells were arranged orderly in control rats. Conversely, ATC rats exhibited slightly swollen skeletal muscle cells, without necrosis and hemorrhage (Figure 5(b)). These results demonstrated the successful establishment of the ATC rat model.

The top 10 candidate miRNAs and lncRNAs following Sankey diagram analysis were examined by RT-qPCR. The results showed that the hsa-miR-24-3p expression was higher, and the KCNQ1OT1 expression was lower in liver tissues of ATC rats than in the control rats, while the expression of other candidate miRNAs and lncRNAs was not significant (Figures 5(c) and 5(d)). Further detection on the VWF protein expression in liver tissues of rats revealed a decline in the liver tissues of ATC rats compared with control rats (Figure 5(e)). Meanwhile, RT-qPCR data presented that the expression of hsa-miR-24-3p was increased in the peripheral blood of ATC patients, while that of KCNQ1OT1 and VWF was decreased compared with healthy individuals (Figure 5(f)). The above data indicated that KCNQ1OT1-hsa-miR-24-3p-VWF ceRNA network was closely related to the ATC development.

## 4. Discussion

The role of lncRNA-miRNA-mRNA axis in the pathogenesis of human diseases has been largely reported and highlighted [18, 19]. This study identified a ceRNA network of KCNQ1OT1, hsa-miR-24-3p, and VWF and confirmed its involvement in the development of ATC. This should be helpful for better understanding of the mechanism of ATC and searching for potential diagnostic targets and treatment options for ATC.

VWF is of great importance in the hemostasis, which can be illustrated by the fact that its deficiency and/or abnormality causes von Willebrand disease (VWD), the most frequent inherited human bleeding disorder [17]. VWF has been reported to be involved in the platelet dysfunction where the increased binding of VWF to platelets may contribute to the platelet dysfunction and platelet clearance [20]. In addition, published data suggested that VWF conferred protection on the fibrinogen from degradation by means of plasmin preserving its clottability in the plasma and its adhesive role in the platelet-rich thrombi [21]. Meanwhile, ATC has been confirmed to be ascribed to the activation of protein C, endothelial glycocalyx disruption, depletion of fibrinogen, and platelet dysfunction [2]. Therefore, VWF may be a key gene involved in the development of ATC.

Further analysis of the present study validated KCNQ1OT1 and hsa-miR-24-3p as the key upstream regulatory factors of VWF, involved in the development of ATC. Consistently, VWF has been well documented to be a downstream target gene of miR-24 in MG-63 and U2OS cells, and the migration-inhibiting activity of miR-24 can be significantly attenuated by the overexpression of VWF [22]. Importantly, the expression of miR-24 is significantly higher in patients with trauma-induced coagulopathy than that in healthy controls, and this high expression is closely correlated to the progression of trauma-induced coagulopathy [23]. In addition, the abnormal expression of lncRNAs has been implicated in the primary immune thrombocytopenia, which is an autoimmune disorder characterized by a decrease in platelets, in which lncRNA function via targeted genes to mediate their functions and consequently affect the development of immune thrombocytopenia [24]. However, the potential role of KCNQ1OT1 in ATC or its related diseases remains unknown. Additionally, the established correlation of KCNQ1OT1 and VWF in the current study requires further investigation due to the little supporting evidence.

Furthermore, the present study revealed that the ceRNA network consisting of KCNQ1OT1, hsa-miR-24-3p, and VWF was implicated in the development of ATC where KCNQ1OT1 competitively bound to hsa-miR-24-3p and caused upregulation of the hsa-miR-24-3p target VWF expression. An increasing number of studies have confirmed that lncRNAs can act as miRNA sponges and thus reduce their regulatory effects on the target mRNAs; for instance, KCNQ1OT1 acts as miR-216b-5p sponge to attenuate its binding ability to ZNF146 and induce upregulation of ZNF146 expression in colorectal cancer cells [25]. In addition, KCNQ1OT1 can competitively target miR-148a-3p and consequently promote the expression of the miR-148a-3p target IGF1R gene in hepatocellular carcinoma cells [26]. KCNQ1OT1 acts as a ceRNA of miR-200a to upregulate the miR-200a downstream forkhead box O3 (FOXO3) expression, thus influencing the pathology of cerebral ischemic stroke [25].

## 5. Conclusion

**Figure 6:**
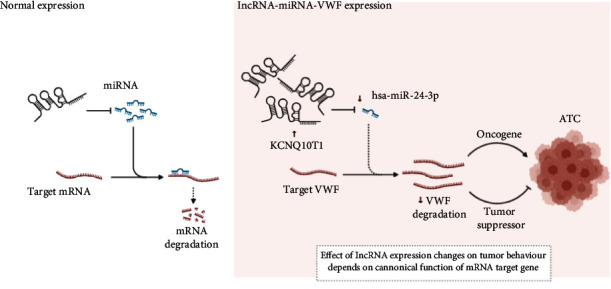
A schematic diagram of the molecular regulation of the KCNQ1OT1-hsa-miR-24-3p-VWF ceRNA network involved in ATC. The upstream KCNQ1OT1 and hsa-miR-24-3p of VWF may be the key factors of the development of ATC. The KCNQ1OT1-hsa-miR-24-3p-VWF ceRNA network may be essential for the development of ATC.

In conclusion, the key findings from this study support a conclusion that VWF and its upstream KCNQ1OT1 and hsa-miR-24-3p might be pivotal factors affecting the development of ATC. Besides, the KCNQ1OT1-hsa-miR-24-3p-VWF ceRNA network can be strongly associated with the development of ATC (Figure 6), which might provide theoretical basis to explore the underlying mechanisms and develop new therapeutic strategies for the prevention and treatment of ATC.

## Data Availability

The datasets generated/analyzed during the current study are available upon request to the corresponding author.

## Abbreviations

ceRNA: Competitive endogenous RNA
ATC: Acute traumatic coagulopathy
lncRNA-miRNA-mRNA: Long noncoding RNA-microRNA-messenger RNA
VWF: Von Willebrand factor
3′UTR: 3′untranslated region
tMCAO: Transient middle cerebral artery occlusion
PT: Prothrombin time
APTT: Activated partial thromboplastin time
HE: Hematoxylin-eosin
cDNA: Complementary DNA
RT-qPCR: Reverse transcription quantitative polymerase chain reaction
NCBI: National Center for Biotechnology Information
BCA: Bicinchoninic acid
PVDF: Polyvinylidene fluoride
ECL: Enhanced chemiluminescence
WT: Wild type
MUT: Mutant type
RIP: RNA binding protein immunoprecipitation
ANOVA: Analysis of variance
BP: Biological process
MF: Molecular function
VWD: Von Willebrand disease
FOXO3: Forkhead box O3.

## Supplementary Materials

Supplementary Materials Supplementary Table 1: primer sequences for reverse transcription quantitative polymerase chain reaction. Supplementary Table 2: predicted upstream miRNAs of VWF. Supplementary Table 3: predicted upstream lncRNAs of VWF. Supplementary Table 4: clinical characteristics of the included ATC patients and healthy individuals.
