# Supplementary material for: Screening of lncRNA-miRNA-mRNA Coexpression Regulatory Networks Involved in Acute Traumatic Coagulation Dysfunction Based on CTD, GeneCards, and PharmGKB Databases
Source: Oxid Med Cell Longev. 2022 Apr 19;2022:7280312. doi: 10.1155/2022/7280312 (PMC9042625; doi:10.1155/2022/7280312)
Supplement: Supplementary Materials — Supplementary Table 1: primer sequences for reverse transcription quantitative polymerase chain reaction. Supplementary Table 2: predicted upstream miRNAs of VWF. Supplementary Table 3: predicted upstream lncRNAs of VWF. Supplementary Table 4: clinical characteristics of the included ATC patients and healthy individuals. [file 7280312.f1.docx]

**SUPPLEMENTARY TABLE 1:** Primer sequences for reverse transcription quantitative polymerase chain reaction

| Gene | Primer sequence |
| --- | --- |
| VWF | Forward: 5’-TCAGTGTCATGATCTGTCCTCC-3’ |
|  | Reserve: 5’-ACACAGGTGAATGTTGTGGGA-3’ |
| KCNQ1OT1 | Forward: 5’-CATCGGTGCCCGTCTGAACAGG-3’ |
|  | Reverse: 5’-TTGCTGGGTAGGAAGAGCTCAG-3’ |
| hsa-miR-24-3p | Forward: 5’-ACACTCCAGCTGGGTGGCTCAGTTCAGCAG-3’ |
|  | Reverse: 5’-CTCAACTGGTGTCGTGGAGTCGGCAATTCAG-3’ |
| β-actin | Forward: 5’-TGTTACCAACTGGGACGACA-3’ |
|  | Reverse: 5’-CTTTTCACGGTTGGCCTTAG-3’ |

**SUPPLEMENTARY TABLE 2:** Predicted upstream miRNAs of VWF

| miRNA | microT | miRWalk | TargetScan | RNA22 | TarBase |
| --- | --- | --- | --- | --- | --- |
| hsa-miR-24-3p | + | + | + | + |  |
| hsa-miR-18a-5p | + | + |  | + |  |
| hsa-miR-17-5p | + | + |  | + |  |
| hsa-miR-19b-1-5p | + | + |  | + |  |
| hsa-miR-19b-3p | + | + |  | + |  |
| hsa-miR-17-3p | + | + |  | + |  |
| hsa-miR-18a-3p | + | + |  | + |  |
| hsa-miR-6796-5p | + | + | + |  |  |
| hsa-miR-3169 | + | + | + |  |  |
| hsa-miR-4437 | + | + | + |  |  |
| hsa-miR-4296 | + | + | + |  |  |
| hsa-miR-589-3p | + | + | + |  |  |
| hsa-miR-6847-5p | + | + | + |  |  |
| hsa-miR-4322 | + | + | + |  |  |
| hsa-miR-6759-5p | + | + | + |  |  |
| hsa-miR-2467-3p | + | + | + |  |  |
| hsa-miR-4674 | + | + | + |  |  |
| hsa-miR-2278 | + | + | + |  |  |
| hsa-miR-3678-3p | + | + | + |  |  |
| hsa-miR-6854-5p | + | + | + |  |  |
| hsa-miR-4265 | + | + | + |  |  |
| hsa-miR-3154 | + | + | + |  |  |
| hsa-miR-6868-5p | + | + | + |  |  |
| hsa-miR-15a-5p | _+_ |  |  | + | + |
| hsa-miR-16-5p | + |  |  | + | + |

**SUPPLEMENTARY TABLE 3:** Predicted upstream lncRNAs of VWF

| miRNAs | lncRNAs |
| --- | --- |
| hsa-miR-24-3p | XLOC_001401, XLOC_006786, MEF2C-AS1, KCNQ1OT1 |
| hsa-miR-18a-5p | RP11-363G2.4 |
| hsa-miR-6796-5p | XLOC_009925, RP11-15H20.6, KCNQ1OT1, CTC-459F4.3, XLOC_004128 |
| hsa-miR-15a-5p | KCNQ1OT1 |
| hsa-miR-18a-5p | CTBP1-AS2, RP11-363G2.4, XLOC_006263 |
| hsa-miR-17-5p | KCNQ1OT1, XLOC_009043 |
| hsa-miR-19b-1-5p | CTBP1-AS2 |
| hsa-miR-19b-3p | KCNQ1OT1, XLOC_013274, RP11-15H20.6 |
| hsa-miR-17-3p | KCNQ1OT1, AC005537.2 |
| hsa-miR-18a-3p | XLOC_012981 |
| hsa-miR-6796-5p | KCNQ1OT1 |
| hsa-miR-3169 | XLOC_000992 |
| hsa-miR-4437 | KCNQ1OT1 |
| hsa-miR-4296 | XLOC_006058 |
| hsa-miR-589-3p | KCNQ1OT1, XLOC_013274, RP11-15H20.6 |
| hsa-miR-6847-5p | RP13-216E22.4 |
| hsa-miR-4322 | KCNQ1OT1 |
| hsa-miR-6759-5p | POLDIP2, RP5-894A10.6, RP11-311C24.1, OIP5-AS1 |
| hsa-miR-2467-3p | XIST, H19, LINC00617 |
| hsa-miR-4674 | XIST |
| hsa-miR-2278 | RP11-228B15.4 |
| hsa-miR-3678-3p | RP11-834C11.4 |
| hsa-miR-6854-5p | XIST |
| hsa-miR-4265 | RP11-977G19.5, RP11-553L6.5 |
| hsa-miR-3154 | SDCBP2-AS1, RP11-379K17.11 RP11-228B15.4 |
| hsa-miR-6868-5p | AC156455.1 |

**SUPPLEMENTARY TABLE 4:** Clinical characteristics of the included ATC patients and healthy individuals

| Clinical characteristics | healthy individuals | ATC patients | *p* value |
| --- | --- | --- | --- |
| Age | 43.27 ± 10.66 | 45.17 ± 10.07 | > 0.05 |
| Gender (male) | 22 | 21 | > 0.05 |
| Blood routine |  |  |  |
| PLT | 157.17 ± 71.79 | 105.37 ± 37.61 | < 0.05 |
| PT | 12.63 ± 1.25 | 15.80 ± 1.21 | < 0.05 |
| Coagulation indicators |  |  |  |
| APTT | 50.13 ± 6.18 | 67.03 ± 4.21 | < 0.05 |
| INR | 1.19 ± 0.21 | 2.24 ± 0.18 |  |
| Six new coagulation and fibrinolysis indexes |  |  | < 0.05 |
| TAT | 2.33 ± 1.07 | 3.51 ± 0.54 | < 0.05 |
| PIC | 1.59 ± 0.53 | 2.68 ± 0.48 | < 0.05 |
| TM | 17.14 ± 3.83 | 25.23 ± 3.34 | < 0.05 |
| t-PAIC | 10.40 ± 0.98 | 3.42 ± 0.43 | < 0.05 |
| FDP | 3.37 ± 1.07 | 5.10 ± 0.55 | < 0.05 |
| D-Dimer | 1.84 ± 0.78 | 2.61 ± 0.26 | < 0.05 |
